# Diagnostic value of virtual autopsy using pm-MRI at 3T on malformed second trimester fetuses vs classic autopsy

**DOI:** 10.1371/journal.pone.0260357

**Published:** 2021-11-29

**Authors:** Adelina Staicu, Camelia Albu, Roxana Popa-Stanila, Cosmina Ioana Bondor, Ioana Cristina Rotar, Florin Stamatian, Daniel Muresan

**Affiliations:** 1 1st Department of Obstetrics and Gynecology, Iuliu Haţieganu University of Medicine and Pharmacy, Cluj‐Napoca, Romania; 2 Department of Pathology, Iuliu Haţieganu University of Medicine and Pharmacy, Cluj‐Napoca, Romania; 3 Centre of Advanced Research Studies, Emergency County Hospital, IMOGEN, Cluj‐Napoca, Romania; 4 Department of Radiology, Iuliu Haţieganu University of Medicine and Pharmacy, Cluj‐Napoca, Romania; 5 Department of Medical Informatics and Biostatistics, Iuliu Haţieganu University of Medicine and Pharmacy, Cluj‐Napoca, Romania; INSERM, FRANCE

## Abstract

**Objective:**

To determine the diagnostic value of virtual autopsy using post mortem-MRI (pm-MRI) at 3Tesla (T) compared to classic autopsy for the confirmation of fetal structural anomalies and secondly to establish which cases of termination of pregnancy would benefit mostly from a virtual autopsy.

**Methods:**

In each of 32 fetuses included in the study, 32 anatomical structures were assessed, after termination of pregnancy in the second trimester. All cases were evaluated by prenatal ultrasonography, virtual autopsy and classic autopsy, and then divided into four groups: Cerebral Group, Cardiac Group, Renal Group and Other Group (miscellaneous group). The concordance of virtual autopsy with classic autopsy was calculated overall and for each group and each structural item. Also, the concordance between the two methods was assessed using a diagnostic error score (DgE_score), calculated as the absolute value of the difference between the number of malformations detected by classic autopsy per case (CA score) and the number of malformations detected at virtual autopsy per case (VA score).

**Results:**

Overall virtual autopsy demonstrated a diagnostic sensitivity (Se) compared to classic autopsy of 67.33% [95% CI 57.28–76.33], with a specificity (Sp) of 98.37% [95% CI 97.33–99.09], a positive predictive value (PPV) of 81.93% [95% CI 71.95–89.52], a negative predictive value (NPV) of 96.49% [95% CI 95.11–97.57] achieving a diagnostic accuracy of 95.31% [95% CI 93.83–96.52]. Overall, no statistic significant correlation was demonstrated between DgE_score and the gestational age of the fetuses or between DgE_score and the weight of the fetuses, but a significant correlation was revealed between the virtual autopsy and classic autopsy score. The diagnostic utility of virtual autopsy using pm-MRI at 3 T as compared to classic autopsy for each category of termination of pregnancy revealed in the Cerebral Group a Se of 80.00% [95% CI 28.36–99.49], with a 96.30% [95% CI 81.03–99.91], a PPV of 80.00% [95% CI 35.75–96.64] a NPV of 96.30% [95% CI 81.81–99.34], with a diagnostic accuracy of 93.75% [95% CI 79.19% to 99.23] and a Cohen’s Kappa coefficient of 0.76 [95% CI 0.4494–1.0765]; in the Renal Group a Se and Sp of 100%, but in the Cardiac Group the Se was only 60.00% [95% CI 26.24–87.84], Sp 75% [95% CI 34.91–96.81], the PPV 75.00% [95% CI 44.92–91.69], NPV 60% [95% CI 38.87–77.96], with a diagnostic accuracy of 66.67% [95% CI 40.99–86.66] and a Cohen’s Kappa coefficient of 0.32 [95% CI -0.07–0.76].

**Conclusions:**

The results support virtual autopsy using pm-MRI at 3T as a reliable alternative to classic autopsy for the non-forensic analysis of second trimester fetuses. Analyzing the diagnostic utility of virtual autopsy using pm-MRI at 3 T for the confirmation of prenatal ultrasound findings in second trimester fetuses as compared to classic autopsy, the best results were obtained in the Cerebral and Renal Group. Reserved results were found in the Cardiac Group. Therefore, for the pregnancies with termination of pregnancy for cerebral or renal abnormalities, virtual autopsy by pm-MRI at 3T can be taken into consideration as a first-line investigation to confirm the prenatal findings.

## 1. Introduction

As technology is becoming more affordable and available in various medical settings, virtual autopsy of small fetuses and children could serve as a reliable alternative in postmortem diagnosis in selected cases [1]. While conventional post mortem methods are customary mandatory, the exact place of virtual autopsy in the clinical diagnosis algorithm is far from being established.

In cases of congenital anomalies ultimately resulting in neonatal demise or that pose significant threat to the quality of life of the child, after accurate prenatal imagistic diagnosis, available options, including termination of pregnancy, should be offered to the parental couple.

As problematic of a decision for the couple it is, classic autopsy may acquire important additional information that completely modifies the prenatal diagnosis in up to one quarter of cases [2, 3]. Considering that typically the final fetal diagnosis is confirmed by classic fetal autopsy obtaining informed parents’ consent for postmortem investigations is valuable as it may influence indirectly the quality of prenatal screening programs and parental couple counseling accuracy [4–8].

If desired, the option of seeing the fetus after birth could aid parents in understanding the cause of fetal loss and confirms the structural defect. However, most often the sight of the deformed fetus can have a severe emotional impact, especially if the patients are not properly counseled. A black-and-white MRI image is more impersonal and therefore less associated with a human person, which may explain the increasing acceptability rate of non-invasive virtual autopsy [9].

In the case of pregnancies that have been terminated medically for karyotype abnormalities, confirmed prenatally by karyotype via amniocentesis, laborious procedures of dissection, staining and microscopic interpretation will most often not provide additional information as compared to an exclusively imagistic investigation [10]. All of these situations might potentially benefit from a rapid virtual autopsy that will confirm/infirm the abnormal prenatal structural findings, and answer any parents’ questions.

In the present study we approached the subject of alternative investigation methods to analyze fetuses resulted from medically terminated pregnancies.

The goal of the study was to determine the diagnostic value of virtual autopsy using post pm-MRI at 3 T compared to classic autopsy for the confirmation of structural anomalies in second trimester fetuses resulted from termination of pregnancy and secondly to establish which cases of termination of pregnancy would benefit mostly from a virtual autopsy.

## 2. Materials and methods

A prospective, longitudinal study was conducted between March 2015-January 2018 in the 1^st^ Clinic of Obstetrics and Gynecology Cluj-Napoca, Emergency County Clinical Hospital Cluj-Napoca, Romania (ECCHCN). The study group enrolled 32 pregnancies with gestational ages ranging from 18 to 23 weeks of gestation (WG). Inclusion criteria for the study groups were singleton evolving pregnancies diagnosed with either chromosomal abnormalities or fetal structural anomalies admitted for termination of pregnancy (indications within the legal framework).

The Standards for Reporting Qualitative Research checklist were used when writing current report [11].

The Ethic Committee of the University of Medicine and Pharmacy Iuliu Haţieganu Cluj Napoca approved the study protocol (124/6.03.2015). Prior to their inclusion in the study all subjects signed the informed consent elaborated according to the World Medical Association Declaration of Helsinki, revised in 2000, Edinburgh.

The ultrasound fetal examination by Voluson E8 Expert machine with transabdominal GE/RAB2-5-D 3D/4D convex probe 1–4 MHz, following the Romanian Society of Obstetrics and Gynecology protocol was used to evaluate each pregnancy.

Following the confirmation of sever fetal anomalies, the genitors were counseled regarding the postnatal prognosis and the impact of the malformations on the extra-uterine life. If opted for, after obtaining the written consent of ECCHCN, termination of pregnancy was performed using prostaglandins, administered locally and orally, according to the internal protocol of the department.

Of the 32 fetuses enrolled, 31 were stillborn. A 23 WG fetus weighing >500g, with a non-viable cardiac malformation was live-born. The neonatology team provided supportive care as per internal protocols for 20 min.

On the same day or the next day, depending on the time of the delivery, the fetuses and the placenta were transported to IMOGEN–the Medical Research Institute within CECHCN, in compliance with to the Human Tissue Act (2004). The fetuses were stored at 4–8°C until the pm-MRI at 3T investigation.

In the above-mentioned center all fetuses were subject first to virtual autopsy and subsequently to classic autopsy in the pathology department.

All information was centralized in a database by a statistician without medical knowledge.

### 2.1 Post mortem 3T MRI examination

A GE Healthcare (DiscoveryTM MR750w GEM) machine with a 3 T magnetic field was used for the MRI examination of the fetuses. The fetuses were scanned in dorsal position, with the head forward using a three-dimensional T2 CUBE protocol (relaxation time 3000, echo time 122, 24x19.2 field of view, slice thickness 1 mm, 0 spacing) and T1 three-dimensional CUBE (relaxation time 683, echo time 18.3, field of view 24x19.2, slice thickness 1 mm, 0 spacing) and 0.5/0.5/0.5mm voxel.

The preexisting sagittal plane scanning available from the GE Healthcare device adapted to their best resolution were applied. The optimum acquisition time was around 15 minutes per sequence. The device station—GE Advantage Workstation (GE Healthcare, Chicago, IL, USA) for image processing and performing volumetric reconstructions was used for visualization.

### 2.2 Image analysis

A radiologist with expertise in pediatric radiology, unbiased by classic autopsy results, analyzed the MRI images acquired beforehand using a standardized protocol. Thirty-two items from nine anatomic segments: nervous, respiratory, cardiac, vascular, renal, digestive segments, facial massive and skeleton were analyzed. For each case, the absence/total number of structural malformations detected per anatomic segment analyzed was recorded; at the end of the examination a final diagnosis including all anomalies was formulated. The images relevant to the diagnosis were attached to individual files.

### 2.3 Postmortem pathologic examination

The pathological examination was performed by two pathologists specialized in fetal pathology according to the autopsy protocol [12] of the French Society of Fetal Pathology, blinded to the virtual autopsy results.

### 2.4 Sample size

Considering alpha error 0.05, fetal structural abnormalities prevalence 0.29 detected by autopsy [13], confidence interval dimension of 0.10 and specificity 0.87 [13] the computed sample size was determined with specificity plot to be approximatively n = 32 [14].

### 2.5 Statistical analysis

For the statistical analysis, SPSS ® (version 15.0, SPSS Inc, Chicago IL USA), Statistica ® (version 8.0, StatSoft Inc, Tulsa OK USA) and Microsoft Excel® 2016 were used. An electronic database has been created that included for every patient: clinical data, ultrasound findings, MRI analysis and pathological findings. A p below 0.05 was considered statistically significant. In order to evaluate the overall diagnostic accuracy of virtual autopsy compared to the accepted gold standard (classic autopsy), sensitivity (Se), specificity(Sp), positive predictive value (PPV), negative predictive value (NPV), diagnostic accuracy and their 95% confidence intervals (CI) were calculated using Dag_stat extension of Microsoft Excel and Interactive statistical page [14]. Cohen kappa coefficient of agreement with the 95% confidence interval and the McNemar test were also used. In addition, in order to highlight the concordance between the two methods, we calculated a diagnostic error score (DgE_score) as the absolute value of the difference between the number of malformations detected by classic autopsy per case (CA score) and the number of malformations detected at virtual autopsy per case (VA score). VA score was calculated for each fetus as the sum of all abnormalities detected from all 32 structure considered to be analyzed by virtual autopsy using pm-MRI at 3 T. CA score was calculated for each fetus as the sum of all abnormalities detected from the same analyzed structures using classic autopsy:

DgE_score=|CAscore–VAscore|

The relationship between two discrete or continuous quantitative variables was evaluated using the correlation coefficient. The averages of the discrete variables were compared using Student t test for paired samples.

In the second part of the study, the cases were divided into four groups according to the prenatally affected system, regardless of the karyotype, into:

- Cerebral Group (CeG)–cerebral anomalies;
- Cardiac group (CaG)–cardiac malformations;
- Renal Group (ReG)–renal malformations and/or early severe olygohydramnios;
- Other Group (OtG) which included fetuses with other rare plurimalformative syndromes (eg, diaphragmatic hernia, sacrococcygeal teratoma) or karyotype abnormalities not found in other groups.

A fetus with multisystemic anomalies was included in all the categories that matched. For example, a case with Trisomy 18 and multiple cerebral anomalies and hydrops was concomitant included in the CeG and OtG.

Se, Sp, PPV, NPV, accuracy with 95% CI, and coefficient of concordance were calculated for each group.

## 3. Results

The total number of structures evaluated was 1024 (32 fetus x 32 items evaluated). The patient’s and fetuses characteristics are depicted in Table 1.

**Table 1:** Characteristics of the study population.

Characteristic	Number
Maternal age	29.90 years (min 24 years, max 36 years min)
Genetic results	9 fetuses (28%) normal karyotype
	13 fetuses (41%) abnormal karyotype (21 Trisomy, 18 Trisomy, Duchenne muscular dystrophy, Autosomal recessive polycystic kidney disease)
	10 fetuses (31%) no genetic test
Mean gestational age of the fetuses	20.5 gestational weeks (min 18 gestational weeks, max 23 gestational weeks)
Mean weight of the fetuses	433.77g (min 155g, max 981g)

102 structures (9.86%) presented structural anomalies confirmed by classic autopsy.

**Fig 1:**
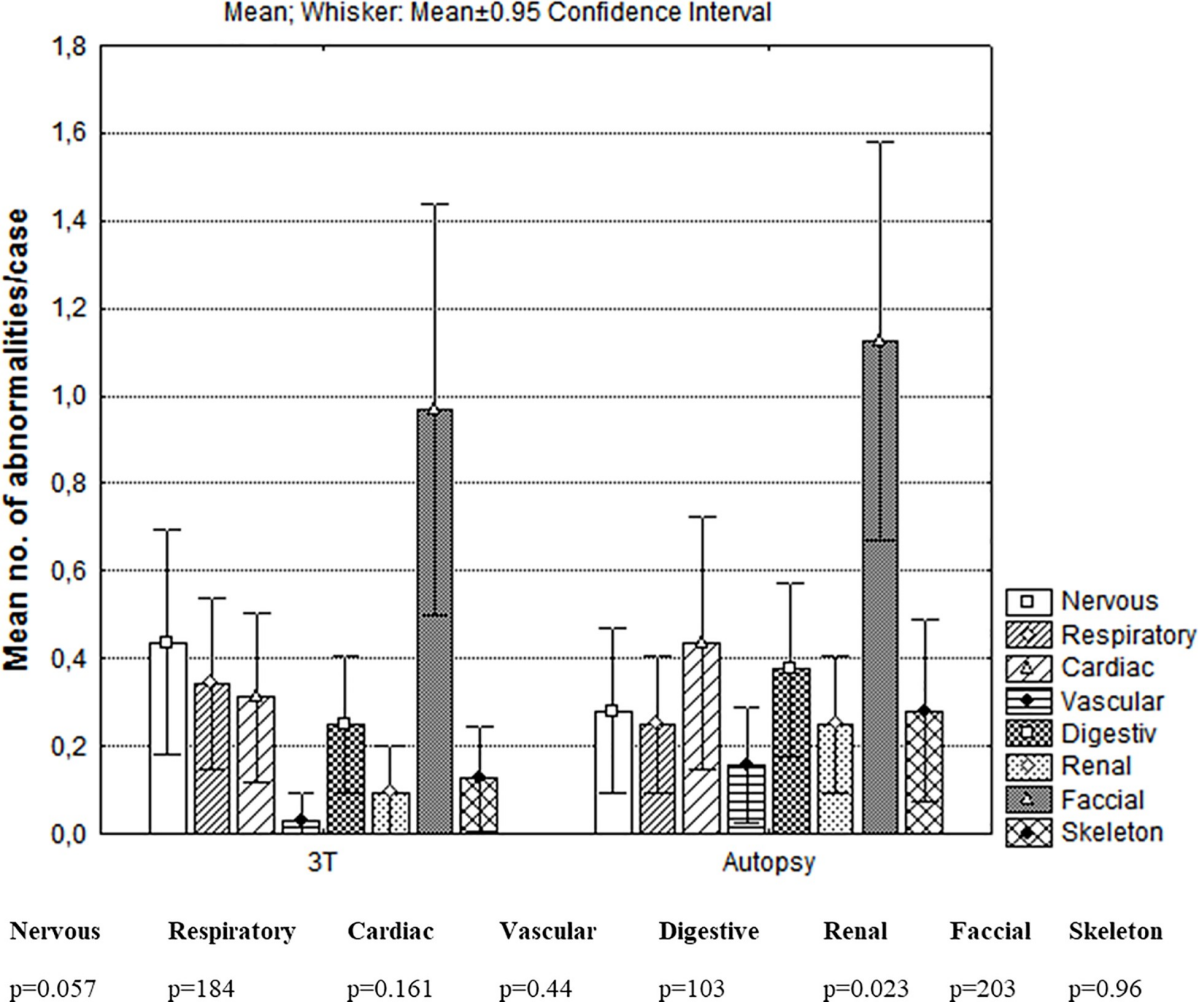
Comparison between the numbers of abnormalities detected per case by virtual autopsy and conventional autopsy considering the anatomic segments. The mean number of abnormalities detected per case using virtual autopsy at 3 T and classic autopsy for each anatomic segment considered was evaluated using p value.

Comparison between the number of abnormalities per case detected by virtual autopsy and classic autopsy considering the anatomic segments is depicted in Fig 1.

When comparing the mean between the numbers of abnormalities detected per case by the two methods, significant differences were found between virtual autopsy at 3T and classic autopsy, in the case of the vascular and renal segments, virtual autopsy detecting fewer abnormalities than autopsy.

Virtual autopsy failed to identify the stenotic pathology of the large vessels, and a corrected transposition of large vessels in a 256 g fetus. However it should be mentioned that in the renal segment, where classic autopsy identified 12 abnormalities in total, 4 of them were described only microscopically, therefore they could not be identified by virtual autopsy at 3 T (2 cases of posterior urethral valve, one renal hypoplasia and one case of undifferentiated external genitalia).

Overall, virtual autopsy demonstrated a diagnostic sensitivity as compared to classic autopsy of 67.33% [95% CI 57.28–76.33], with a specificity of 98.37% [95% CI 97.33–99.09], a PPV of 81.93% [CI 95% 71.95–89.52], a NPV of 96.49% [95% CI 95.11–97.57] achieving a diagnostic accuracy of 95.31% [95% 93.83–96.52], Cohen’s kappa coefficient of correlation was 0.71, revealing a substantial agreement between the two methods, also confirmed by the McNemar test = 6.75 (p = 0.0094).

The diagnostic accuracy of the virtual autopsy using pm-MRI as compared to the autopsy reported to specific anatomic segments is shown in Table 2.

**Table 2:** Statistical analysis of the diagnostic utility of virtual autopsy at 3 T compared to the classic autopsy related to the fetal anatomical segments evaluated by standard protocol.

Anatomic segment Number of structure evaluated (N) = 32 fetuses x structures evaluated per segment	Sensitivity (%) [CI 95%]	Specificity (%) [CI 95%]	PPV (%) [CI 95%]	NPV (%) [CI 95%]	Accuracy (%) [CI 95%]	Cohen’s Kappa [CI 95%]	Frequency of malformations (%) [CI 95%]	McNemar test p	Structures Evaluated per segment
Nervous system N = 192	88.89%	96.72%	57.14	99.44%	96.35%	0.88	4.69%	1	cerebral hemispheres, ventricular system, thalamus, basal bodies, corpus callosum, cerebellum
	[51.75–99.72]	[93.00–98.79]	36.99–75.17]	[96.54–99.91]	[92.63–98.52]	[0.72.33–1.04]	[2.17–8.71]		
Respiratory system N = 96	100%	96.59%	72.73%	100%	96.88%	0.82	8.33%	0.08	trachea, bronchi, lungs
	[63.06–100.00]	[90.36–99.29]	[46.72–89.02]		[91.14–99.35]	[0.633–1.0169]	[3.67–15.76]		
Cardiac system N = 96	64.29%	98.78%	90.00%	94.19%	93.75%	0.58	14.58%	1	situs solitus, four-chamber image, atrio-ventricular valve appearance
	[35.14–87.24]	[93.39–99.97]	[55.24–98.50]	[88.91–97.04]	[86.89–97.67]	[0.3530–0.8165]	[8.21–23.26]		
Vascular system N = 128	20.00%	100%	100%	96.85%	96.88%	0.32	3.91%	0.04	large vessels at the base of the heart, venous return, aortic arch, descending aorta
	[0.51–71.64]	[97.05–100.00]	96.85%	[95.20–97.95]	[92.19–99.14]	[-0.1559–0.8050]	[1.28–8.88]		
Digestive system N = 224	100%	97.35%	37.50%	100%	97.40%	0.52	1.56%	0.02	appearance of the diaphragm, integrity of the abdominal wall, esophagus, stomach, liver, spleen, pancreas
	[29.24–100]	[93.93–99.14]	[20.17–58.76]		[94.03–99.15]	[0.1887–0.8911]	[0.32–4.50]		
Renal system N = 96	58.33%	98.81%	87.50%	94.32%	93.75%	0.66	12.50%	0.102	kidneys, bladder and genitals
	[27.67–84.83]	[93.54–99.97]	[48.50–98.11]	[89.47–97.01]	[86.89–97.67]	[0.4204–0.9129]			
Facial massive N = 128	90.32%	92.78%	80.00%	96.77%	92.19%	0.79	24.22%	0.205	palate, mandible, ears, eyes
	[74.25–97.96]	[85.70–97.05]	[66.00–89.18]	[91.09–98.88]	[86.10–96.19]	[0.6757–0.9165]	[17.09–32.58]		
Skeleton N = 64	33.33%	98.18%	75.00%	90.00%	89.06%	0.41	14.06%	0.05	anomalies of closure and shape of the column, the presence / absence and anomalies of limbs
	[7.49–70.07]	[90.28–99.95]	[25.88–96.26]	[84.99–93.47]	[78.75–95.49]	[0.0651–0.7559]	[6.64–25.02]		

**Fig 2:**
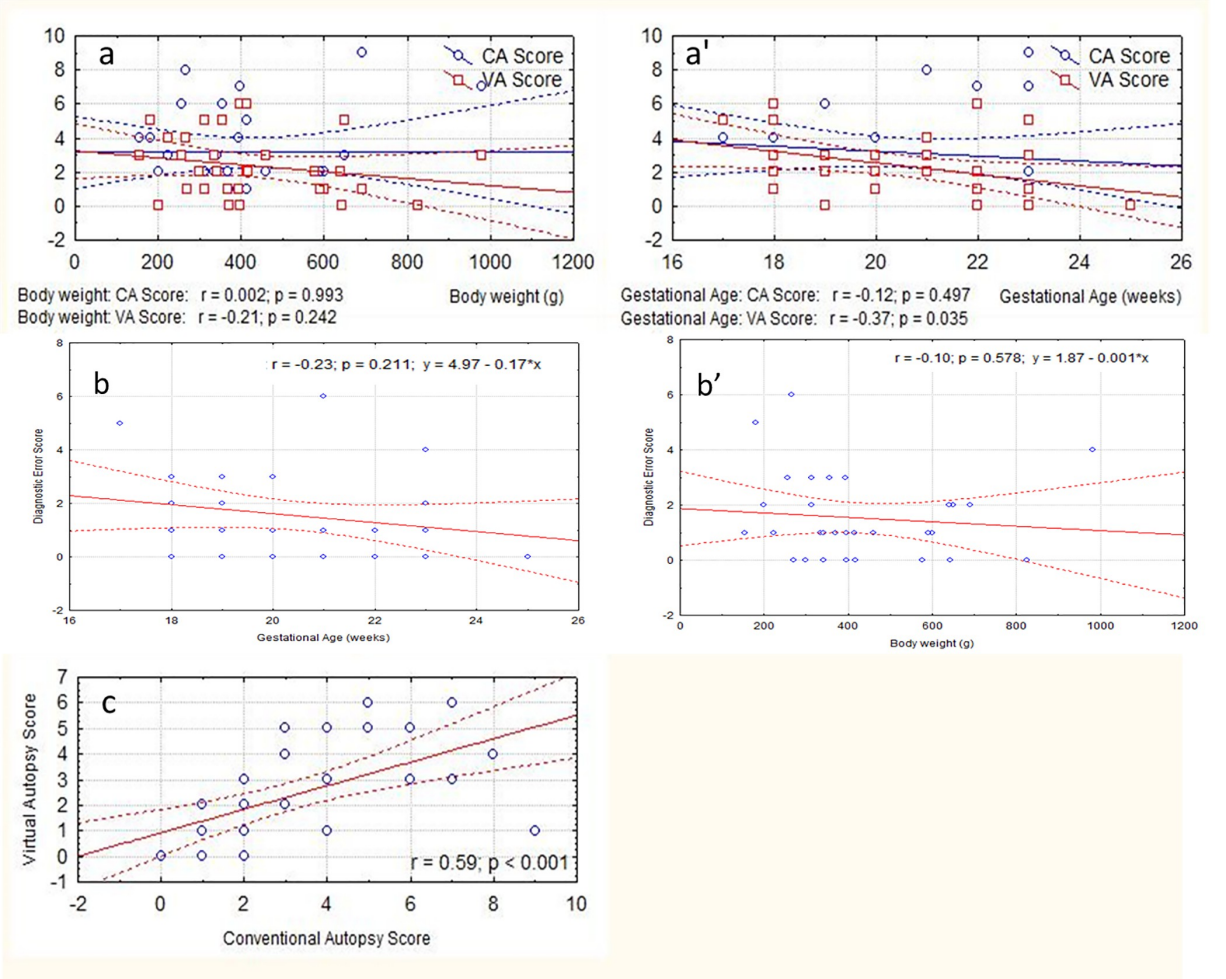
Correlation between virtual autopsy score (VA score), classic autopsy score (CA score) and various parameters. Linear regression line and its 95% confidence interval, Spearman correlation coefficient (r) and significant (p). a) correlation between CA and VA score with fetal body weight; a’) correlation between CA and VA score with gestational age; b) correlation between diagnostic error score (DgE_score) with fetal body weight; b’) correlation between DgE_score with gestational age; c) correlation between VA using pm-MRI at 3T diagnostic score with CA diagnostic score.

In the current study, the overall CA and VA score was not correlated with the fetus body weight (Fig 2A), but overall VA score was positive correlated with the gestational age of the fetuses, the number of anomalies detected per case by virtual autopsies increasing with gestational age (Fig 2A’).

Overall no statistical significant correlation was found between DgE_score and the gestational age of the fetuses or between DgE_score and the weight of the fetuses (Fig 2B and 2B’). Still, between the CA and VA score was a significant positive correlation (Fig 2C).

Taken into account each anatomic segment studied, DgE_score was negative correlated with the gestational age of the fetuses regarding the evaluation of the cerebral segment. Also, DgE_score was positive correlated with the weight of the fetuses in the cardiac segment.

The statistical analysis regarding the diagnostic DgE_score on each anatomic segment and the relation with the gestational age and body weight of the fetuses is depicted in detail in Table 3.

**Table 3:** Correlation between diagnostic score (DgE_score) on each anatomic segment and gestational age and fetal body weight.

	Gestational age (weeks)		Body weight (g)	
	Coefficient of correlation	p	Coefficient of correlation	P
Nervous	-0.44	**0.013**	-0.33	0.067
Respiratory	-0.31	0.089	-0.22	0.230
Cardiac	0.24	0.194	0.45	**0.010**
Vascular	0.14	0.439	0.24	0.180
Renal	-0.24	0.185	-0.24	0.178
Digestive	0.06	0.726	0.04	0.831
Facial	-0.18	0.318	-0.31	0.088
Skeleton	-0.02	0.930	-0.04	0.822

### 3.2 Diagnostic utility of virtual autopsy using pm-MRI at 3T for the confirmation of each category of termination of pregnancy in second trimester fetuses as compared to classic autopsy

In the second part of the present research, we aim to analyze the performance of virtual autopsy as compared to classic autopsy for each category of termination of pregnancy.

The diagnostic utility of virtual autopsy using pm-MRI at 3 T as compared to classic autopsy for each category of termination of pregnancy is depicted in Table 4. The prenatal imaging findings and genetic testing are described in Table 5.

**Table 4:** Diagnostic utility of pm-MRI 3 T compared to conventional autopsy, considering the prenatal indication for therapeutic interruption of pregnancy.

Group Number of cases Average gestational weeks (WG) Average weight	Sensitivity (%)	Specificity (%)	PPV (%)	NPV (%)	Accuracy (%)	Cohen’s Kappa
	[CI 95%]	[CI 95%]	[CI 95%]	[CI 95%]	[CI 95%]	[CI 95%]
Cerebral 9 cases 20.78 WG (SD ± 0.73) 387g (SD ± 64,732)	80.00% [CI 95% 28.36–99.49]	96.30% [CI 95% 81.03–99.91]	80.00% [CI 95% 35.75–96.64]	96.30% [CI 95% 81.81–99.34]	93.75% [CI 95% 79.19% to 99.23]	0.76
						[CI 95% 0.4494–1.0765]
Cardiac 8 cases 20.5 WG(SD ±0.729) 462g (SD ±236)	60.00% [CI 95% 26.24–87.84],	75% [CI 95% 34.91–96.81]	75.00% [CI 95% 44.92–91.69]	60% [CI 95% 38.87% to 77.96]	66.67% [CI 95% 40.99–86.66]	0.32
						[CI 95% -0.07–0.76].
Renal 5 cases 21.5WG(SD ±0.619) 392.5g (SD ±62.88)	100%	100%	100%	100%	100%	1
Other 13 cases 19.85 WG (SD ±0.587) 412.85g (SD ±50.567)	76.92%[CI 95% 60.67–88.87]	98.41% [CI 95% 96.57–99.41]	83.33% [CI 95% 68.95–91.85]	97.63% [CI 95% 95.87–8.65]	96.39% [CI 95%, 94.12–97.97]	0.71

**Table 5:** Description of fetal anomalies detected by ultrasound in every group included in the study.

Group	No of cases. Gestational age. Weight	Karyotype/other genetic test	Prenatal findings	Post mortem findings	
				Pm-MRI	Autopsy
Cerebral (CeG)	1.23WG 691g	18 Trisomy	Fetal hydrops, cystic hygroma, ventriculomegaly, partial agenesis of corpus callosum, arthrogryposis, congenital clubfoot and hydramnios.	Scalp with massive edema; posterior cervical cystic hygroma. Partial agenesis of corpus callosum with present genu and body. Germinal matrix cysts and hemorrhage. Abnormal sylvian fissure opercularization; Irregular insular surface. Facial dysmorphism. Cleft palate. Tracheal compression. Pulmonary hypoplasia. Anasarca. Arthrogryposis. bilateral clubfoot	Scalp shows massive edema.Partial agenesis of corpus callosum. Intraparenchymal hemorrhage. Facial dysmorphism.Cleft palate. Partial agenesis of corpus callosum.Arthrogryposis. Bilateral congenital clubfoot. Pulmonary hypoplasia. Interventricular septum shows defect shows perimembranous ventricular defect. Anasarca.
	2. 22 WG 600g	Normal	Dandy Walker malformation	Dandy Walker malformation	Dandy Walker malformation. Right ventricle hypoplasia
	3. 21 WG 223g	Normal	Agenesis of corpus callosum, fetal non immune hydrops	Lateral ventricles morphology suspected of lobar holoprosencephaly. Right subventricular cyst (probably ischemic). Cerebellum with a smaller transverse diameter than the rest of fetal brain biometrics. Hemochromatosis, bilateral pulmonary hypoplasia, bilateral renal ectopia. No urinary bladder visible	Primary hemochromatosis, bilateral pulmonary; hypoplasia, hypoplasia with renal ectopia; Bilateral, extensive autolysis of the brain.
	4.19 WG 200g	21 Trisomy	Bilateral ventriculomegaly	None	Posterior urethral valves in the proximal third of the urethra detected microscopically, specific facial dysmorphism
	5.23 WG 643g	21 Trisomy	Limb anomalies; ventriculomegaly	none	none
	6.20 WG 393g	Not tested	Thoracolumbar spina bifida, Arnold–Chiari II malformation, pyelectasis	Arnold-Chiari II malformation	Arnold-Chiari II malformation, craniofacial dysmorphism, pielocalicial dilatation
	7.21 WG 265 g	Not tested	Holoprosencephaly, renal hypoplasia, VSD, arthrogryposis	Holoprosencephaly, focal cortical polymicrogyria, ectopic left kidney, pulmonary hypoplasia, arthrogryposis, dolichocephaly	Holoprosencephaly middle interhemispheric variant or synthelencephaly, focal cortical polymicrogyria, renal hypoplasia, ectopic left kidney, pulmonary hypoplasia, hepatic hypoplasia, hypertelorism, arthrogryposis, dolichocephaly
	8. 18 WG 155 g	Not tested	Agenesis of corpus callosum with ventriculomegaly, anomalies of the spine with head hyperflexion	Lisencephaly, retroflexion of the spine, arthrogryposis	Lisencephaly, classic form arthrogryposis, the head and neck are fixed in extreme retroflexion, undifferentiated external genitalia
	9.18 WG 313g	Not tested	Agenesis of corpus callosum, hydrocephaly, ductus venosus agenesis, single umbilical artery, clenched hands	Corpus callosum agenesis, hydrocephaly ductus venous agenesis, esophageal atresia craniofacial dysmorphism.	Polymalformed fetus with venous duct agenesis with extrahepatic anastomosis,corpus callosum agenesis, hydrocephaly, esophageal atresia type C, craniofacial dysmorphism.
Cardiac (CaG)	1.18 WG 180 g	Turner Syndrome	Cervical cystic hygroma. Bilateral pleural collection. Massive subcutaneous thoracal and abdominal. VSD. Unique umbilical artery.	Cervical cystic hygroma, interventricular septal defect, lungs hypoplasie,renal fusion	cervical cystic hygroma, interventricular septal defect, renal fusion
	2.20 WG 334 g	21 Trisomy	AVSD	Atrioventricular canal. Facial dysmorphism	Atrioventricular canal. Facial dysmorphism
	3.23 WG 590 g	Normal	Aortic stenosis with left ventricle fibroelastosis.	Myocardial hypertrophy	Endocardial fibroelastosis, secondary myocardial hypertrophy, functional aortic stenosis
	4.20 WG 414 g	Normal	VSD; transposition of great vessels	ASD, VSD, riding aorta, Pulmonary stenosis, myocardial hypertrophy, lungs hypoplasie	Fallot pentalogy, hepatic ductal malformation
	5.22 WG 577g	21 Trisomy	AVSD	VSD, facial dysmorphism	VSD, facial dysmorphism
	6.23 WG 981g	Not tested	Ebstein tip D (Carpentier) anomaly without pulmonary artery stenosis	Complex cardiac anomaly and megaencephaly	Ebstein type D anomaly (Carpentier) without pulmonary artery stenosis, megalencephaly and syndactyly fingers 2 + 3 bilateral lower limbs
	7.18 WG 356 g	21 Trisomy	AVSD	Atrioventricular canal,	Facial dysmorphism, atrioventricular canal, posterior urethral valve detected microscopically
	8.21 WG 265 g	Not tested	Holoprosencephaly, renal hypoplasia, VSD, arthrogryposis	Holoprosencephaly, focal cortical polymicrogyria, ectopic left kidney, pulmonary hypoplasia, arthrogryposis, dolichocephaly	Holoprosencephaly middle interhemispheric variant or synthelencephaly, focal cortical polymicrogyria, renal hypoplasia, ectopic left kidney, pulmonary hypoplasia, hepatic hypoplasia, hypertelorism, arthrogryposis, dolichocephaly
Renal (ReG)	1.23 WG 651g	ARPKD	Bilateral cystic renal dysplasia, severe oligohydramnios	Bilateral multicystic renal dysplasia, pulmonary hypoplasia, facial dysmorphism, bicorn uterus.	Bilateral multicystic renal dysplasia, pulmonary hypoplasia, facial dysmorphism, bicorn uterus
	2. 23 WG 371g	Normal	Severe oligohydramnios, empty urinary bladder, non-visible left kidney	Dilated colon and rectum	Hirchsprung’s disease pancreatic heterotopia in the gastric wall.
	3. 22 WG 397g	Normal	Bilateral renal dysplasia, transonic 4 cm diameter tumor at the sacrococcygeal level	Multicystic single ectopic kidney.Duplicate ureter. Bladder ptosis.Facial dysmorphism. Pulmonary hypoplasia.	Pelvic floor defect with bladder ptosis. Cystically dilated bladder. Dysplastic kidney multichystic produced in an obstructive context on a single ectopic kidney. Incomplete duplicate ureter. Potter syndrome.
	4. 21 WG 459 g	Not tested	Right renal tumor with important mass effect, oligohydramnios	Bilateral multichystic renal dysplasia and pulmonary hypoplasia	Bilateral multichystic renal dysplasia and pulmonary hypoplasia
	5. 19 WG 256g	Not tested	Oligohydramnios, bilateral renal agenesis	Renal aplasia, pulmonary hypoplasia, facial dysmorphism	DiGeorge Syndrome:thymus aplasia, renal aplasia, anal atresia, pulmonary hypoplasia, left bronchial isomerism, facial dysmophism (lower inserted ears, microretrognathia), hemivertebra. atrioventricular septal defect, endocardial fibroelastosis and corrected transposition of large vessels
Other (OtG)	1. 18 WG 641g	Not tested	Non-immune fetal hydrops. Femur length corresponding to 18 weeks of gestation	Hydrops, ventriculomegaly	Severe hydrops, endocardial fibroelastosis, intestinal malrotation.
	2.18 WG 414g	Not tested	Severe non-immune fetal hydrops. Oligoamnios	Fetal hydrops. Pulmonary hypoplasia. Renal agenesis Congenital clubfoot. Facial dysmorphism.	Fetal hydrops. Pulmonary hypoplasia. Renal agenesis Congenital clubfoot. Facial dysmorphism (Facies Potter).
	3. 18 WG 369g	Normal	Fetal diaphragmatic hernia. Severe oligoamnios.	Severe congenital diaphragmatic left hernia. Lungs hypoplasia	Severe congenital diaphragmatic left hernia. Lungs hypoplasia. Preductal aorta stenosis.
	4. 23 WG 825g	Normal	Sacro-coccygeal teratoma of 63/53 mm, with mixed structure, with moderate vascularization. Both kidneys visible with 6 mm pelvis	Sacrococcygeal teratoma	Mature sacrococcygeal teratoma, type I
	5.18 WG 271g	Not tested	Fetus with biometrics corresponding for 12 S + 4Z. Gastroschizis.	Gastroschisis	Gastroschisis
	6.19 WG 410g	Duchenne muscular dystrophy	No structural anomalies	Facial dysmorphism	Facial dysmorphism
	7.21 WG 417g	21 Trisomy	No structural anomalies	Facial dysmorphism	Facial dysmorphism
	8. 20WG 364g	21 Trisomy	No structural anomalies	None	none
	9. 22 WG 397g	Normal	Non-immune fetal hydrop	Hydrops. No structural abnormalities detected	Hydrops
	10. 20 WG 312g	21 Trisomy	No structural anomalies	Facial dysmorphism	Facial dysmorphysm
	11. 19 WG 341g	21 Trisomy	No structural anomalies	Facial dysmorphism. Cortical polymicrogyria	Facial dysmorphism. Cortical polymicrogyria
	13.23WG 691g	18 Trisomy	Fetal hydrops, cystic hygroma, ventriculomegaly, partial agenesis of corpus callosum, arthrogryposis, congenital clubfoot and hydramnios.	Scalp with massive edema; posterior cervical cystic hygroma. Partial agenesis of corpus callosum with present genu and body. Germinal matrix cysts and hemorrhage. Abnormal sylvian fissure opercularization; Irregular insular surface. Facial dysmorphism. Cleft palate. Tracheal compression. Pulmonary hypoplasia. Anasarca. Arthrogryposis. bilateral clubfoot	Scalp shows massive edema.Partial agenesis of corpus callosum. Intraparenchymal hemorrhage. Facial dysmorphism.Cleft palate. Partial agenesis of corpus callosum.Arthrogryposis. bilateral congenital clubfoot. Pulmonary hypoplasia. Interventricular septum shows defect shows perimembranous ventricular defect. Anasarca.
	14.18 WG 180 g	Turner Syndrome	VSD, fetal hydrops	Cervical cystic hygroma, interventricular septal defect, lungs hypoplasie, renal fusion	Cervical cystic hygroma, interventricular septal defect, renal fusion

ASD = atrial septal defect, VSD = ventricular septal defect, AVSD = atrioventricular septal defect, Autosomal recessive polycystic kidney disease = ARPKD.

**Fig 3:**
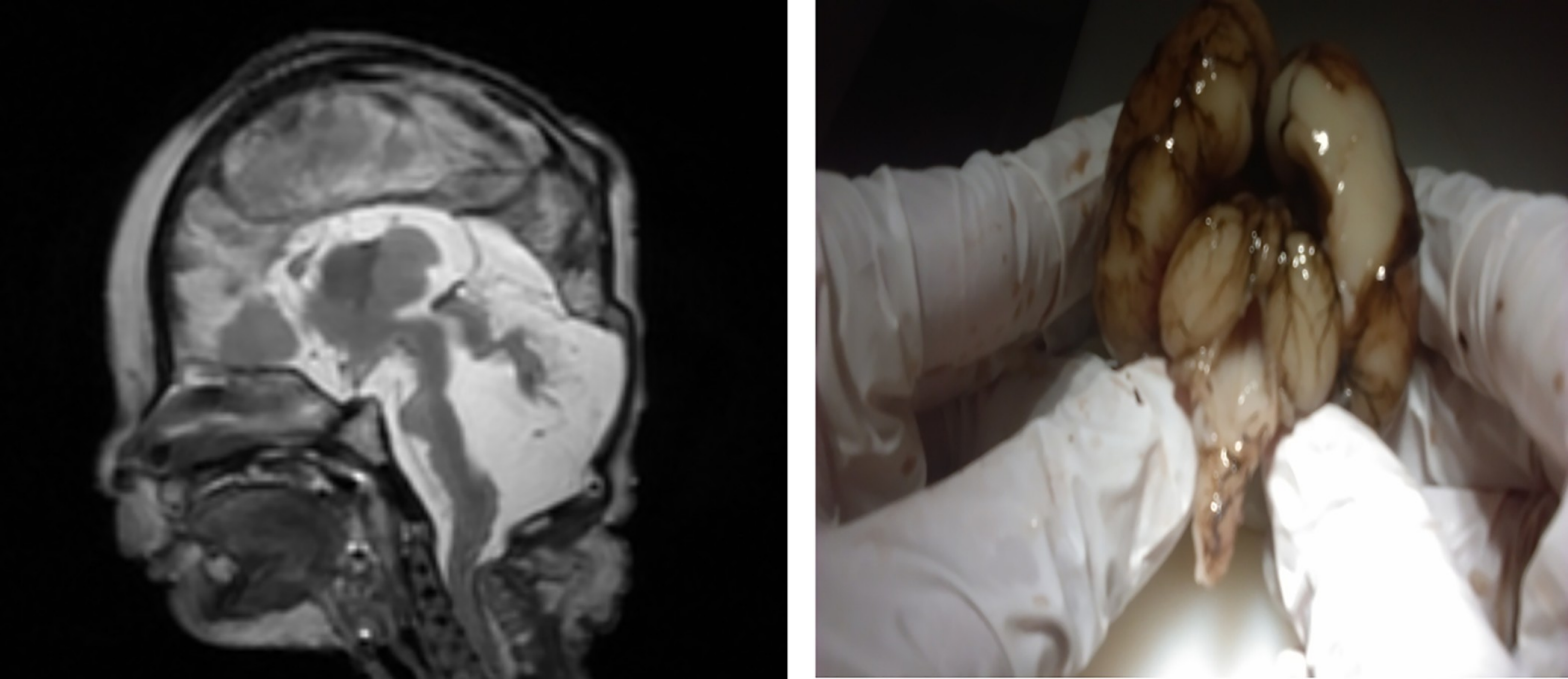
Comparison between post mortem MRI at 3T, T2 WI and conventional autopsy of a 22 weeks of gestation fetus with Dandy Walker malformation. The images depict enlarged posterior fossa, cystic dilatation of the fourth ventricle extending posteriorly, and vermis hypoplasia.

In the **Cerebral Group** (CeG), although the diagnostic concordance between virtual autopsy and classic autopsy was very good, two presumable ultra-sonographic diagnoses of ventriculomegaly in a 19 GW fetus with confirmed trisomy 21 and respectively a 23 WG polymalformed fetus were not confirmed by virtual autopsy, nor by classic autopsy. Also, the ultrasound diagnosis of corpus callosum agenesis in a 21 WG fetus with non-immune hydrops was confirmed only by virtual autopsy. The classic autopsy was not able to confirm the diagnosis due to intense autolysis changes that made the evaluation of the fetal brain impossible. An example of diagnostic concordance between virtual autopsy and classic autopsy in the CeG is render in Fig 3. Also, virtual autopsy was able to detect two cases of polymicrogyria (Case 8 in CeG and case 10 in OtG) that was confirmed by classic autopsy.

The **cardiac abnormalities** from the CaG described by prenatal ultrasound were confirmed by virtual autopsy in 5 of the 8 cases, but classical autopsy confirmed 7 of the abnormalities described by ultrasound. Neither pm-MRI nor autopsy confirmed a ventricular septum defect (VSD) in a fetus of 21 WG.

Both virtual autopsy and classic autopsy confirmed 5 of the 6 renal abnormalities described by prenatal ultrasound in the ReG. In one case where prenatally ultrasound suspected a renal cystic dysplasia, virtual autopsy identified marked dilatation of the colon and rectum with normal kidneys and microscopy confirmed the diagnosis of Hirschsprung disease. Therefore the statistical analysis showed a perfect agreement between the two methods of post-mortem evaluation, with a sensitivity and specificity of the pm-MRI 3T as compared to the classic autopsy of 100%.

**Fig 4:**
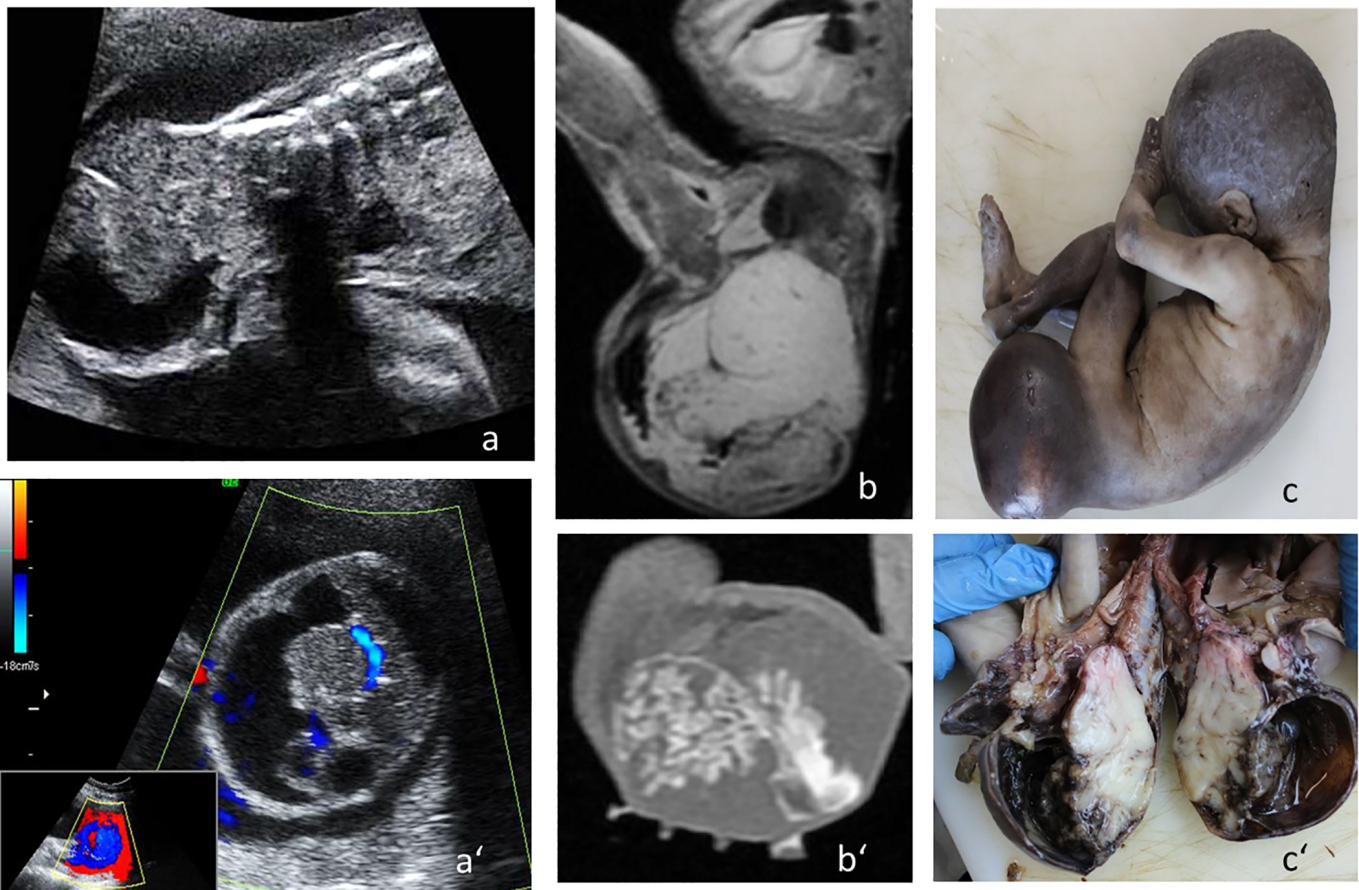
22 weeks of gestation fetus diagnosed prenatally with a), a’) fast growing sacroccigian teratoma. b), b’) pm-MRI at 3 T with injection of gadolinium substance depicting moderate vascularization in the tumor, T2 and T1 WI, sagittal section c),c’) confirmation of the imaging findings by conventional autopsy.

**Fig 5:**
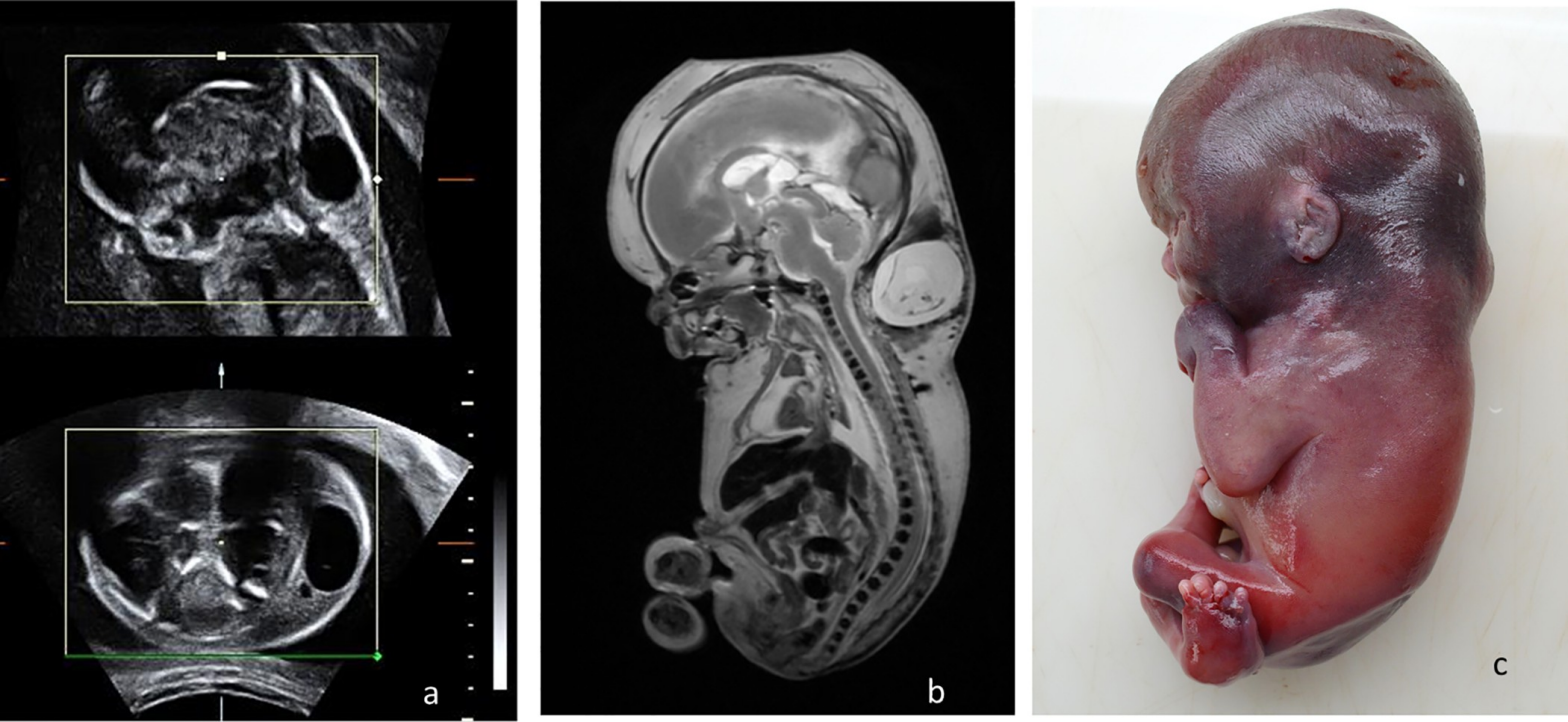
23 weeks of gestation plurimalformed fetus diagnosed prenatally with 18 trisomy. a) prenatal ultrasonography; b) pm-MRI at 3 T, T2 WI sagittal section, demonstrating hydrops fetalis, hygroma, partial corpus callosum agenesis and limbs malformations; c) macroscopic examination of the fetus.

Examples of diagnostic concordance between virtual autopsy and classic autopsy in identifying prenatal findings are rendered in Figs 4 and 5.

## Discussion

The present study demonstrates that virtual autopsy using pm-MRI at 3T allows a very good characterization of the anatomical structures in second trimester fetuses resulted from termination of pregnancy, with an average weight of 433.77g and a relatively small gestational age (18–23 WG). We found substantial agreement between the two methods evaluated by Cohen’s kappa coefficient = 0.71 and Spearman correlations (r = 0.059, p <0.001).

Moreover, the diagnostic accuracy of virtual autopsy using pm-MRI at 3 T compared to classic autopsy was very good (95.31%); still it is debatable if the sensitivity obtained is sufficient for its implementation in the clinical practice.

However, it should be taken into account that the study group consisted only of fetuses resulting from termination of pregnancy for congenital malformations and that the fetuses had a relatively small gestational age; all cases being evaluated by a complete virtual autopsy and classic autopsy. Thus, the results obtained can be considered closer to the true diagnostic reliability of the post-mortem magnetic resonance, than the results of other studies that have studied the diagnostic accuracy of virtual autopsy at 3T but have not included so many structural anomalies [15, 16]. Also blinding both radiologist and pathologist, the diagnostic accuracy of virtual autopsy obtained can be considered the actual diagnostic value at a given time of pm-MRI and not the results of a learning curve in progress for the radiologist.

At the same time, the increased prevalence of malformations, PPV and NPV obtained in our study, rather reflects the population of a tertiary reference center and not the low risk population. These results may recommend virtual autopsy at 3T as an adjuvant, reliable method, of the accepted standard method and possibly as an alternative method, if the parental couple refuses classic autopsy.

The best results were obtained for the evaluation of the nervous segment, where virtual autopsy showed an increased sensitivity and specificity; the data being similar to those obtained by other researchers [17]. Also, considering that most of the brain abnormalities described were severe, characterized by massive destruction of brain tissue (Dandy Walker malformation, corpus callosum agenesis and holoprosencephaly), abnormalities difficult to confirm by classical postmortem evaluation methods, virtual autopsy proved to bring valuable insight. Benefiting from maintaining the normal level of cerebrospinal fluid, virtual autopsy provided a detailed description of the anatomical structures of the brain and was able to confirm the ultrasound diagnosis.

Another malformation that is frequent and that may be difficult to identify at 21 weeks is polymicrogyria [18]. However, virtual autopsy at 3 T was able to accurately detect it even when termination of pregnancy is performed because of other severe malformation, making it a reliable method in the diagnosis process which offers relevant data to expecting parents’ decisional approach.

Pm-MRI had limitations while examining the cardiovascular system: in our study we have reported a low sensitivity of only 64.29%, with a specificity of 98.78% for the cardiac segment and a sensitivity of 20%, with a specificity of 100% for the vascular segment. However, the results obtained are consistent with those previously published in literature [19, 20]. Thayyil recommends that diagnostic accuracy of pm-MRI for cardiac assessment can be improved by three methods: by adding 3D techniques, by secondary image revision by a cardiovascular pediatric radiologist or by using a higher magnetic field [20–22]. Another explanation for the results obtained for the cardio-vascular system may be our incipient experience regarding the aspect of fetal cardiac MRI at 3T.

Pm-MRI at 3 T should nevertheless be regarded as a potential tool to assess cardiac segment, as new studies arise with promising good results in identifying with high specificity the nonseptal cardiac in fetuses weighing more than 100 g, with low degree maceration [23].

The current research reported a high sensitivity for the detection of digestive system abnormalities, although the literature describes the imaging method as deficient for the evaluation of the digestive system [24]. The results obtained can be explained by the small number of digestive malformations existing in the studied group (frequency = 1.56%) and by the fact that intestinal malrotations were not detected at the classic autopsy, as it is known that this type of anomaly is systematically omitted by post mortem imaging investigations [24].

Pm-MRI has shown good sensitivity and specificity for describing facial dysmorphic abnormalities, but imaging is not absolutely necessary, as these characters can be easily described through macroscopic inspection.

For the evaluation of the renal segment pm-MRI 3T obtained a sensitivity of 58.33% with a specificity of 98.81%, results consistent with other studies in the literature [23, 24].

In the current study, except for the cardiac abnormalities, the diagnostic accuracy of virtual autopsy did not depend on the fetus weight after 18 WG. In contrast the paper publishes by Kang et al finds the performance of pm-MRI dependent of the gestational age [13]. This may be explained by the higher number of cases less than 20 weeks of gestation included by the above mentioned group, therefore the error decreased with increasing gestational age.

Taking into account the four groups of recommendations for termination of pregnancy, the best match between the two methods of post-mortem fetal evaluation was found in the Cerebral Group. Therefore we can stipulate that for a fetus with a prenatally known cerebral abnormality, virtual autopsy by pm-MRI can quickly identify cerebral abnormalities with an accuracy of approximately 93.75%. By contrast a classic autopsy would require a longer period for tissue fixation, special techniques and specialized medical personnel.

The diagnostic accuracy obtained for describing cerebral abnormalities detected by prenatal ultrasound in our study, encourages us to recommend virtual autopsy by pm-MRI at 3T as a first-line method of post-mortem diagnosis of cerebral abnormalities, in fetuses between 18–23 WG.

The same encouraging results were obtained in the Renal Group, where virtual autopsy by pm-MRI and classic autopsy had an excellent concordant result, which makes us assert that pm-MRI can be offered as a noninvasive alternative method that can confirm the polycystic kidney syndromes or the renal agenesis that would require the therapeutic termination of pregnancy for renal reasons.

However, it must not be disregarded that in the population considered in the study, an important number of renal anomalies were detected only by microscopy, for example posterior urethral valve and renal hypoplasia. Pm-MRI images which are effective in detecting subtle renal anomalies compared to microscopy were obtained in previous research exclusively using a higher magnetic field like 7T [25]. Another solution to improve the results of pm-MRI at 3 T image interpretations would be the development of scale nomograms regarding the normal and pathological appearance of various organs at a certain gestational age.

Even in the Other Group, pm-MRI obtained a very good diagnostic accuracy as compared to classic autopsy, underlining the potential of pm-MRI to document rare anomalies with imaging that may be used for the efficient evaluation of subsequent cases.

Moreover, the high NPV and the PPV can recommend pm-MRI as a triage method, being able to excellently discern between normal and abnormal organs in order to guide a difficult dissection or to guide a targeted biopsy within minimally invasive autopsies [26].

However, the results obtained in the Cardiac Group cannot yet recommend pm-MRI as an alternative method of virtual autopsy.

The post-mortem diagnoses reported in this study were based on the cumulative experience of pediatric imaging, prenatal ultrasound, fetal anatomy and the intense study of the literature on the post-mortem imaging related to the presence of blood clots, air from the autolysis process and sedimentation [27–30].

An obvious limitation of the present research is the small number of cases included. Only 32 cases could be properly documented, due to the reduced availability of the scanner during normal working hours, and the availability of technicians in the field.

From our point of view, at the moment pm-MRI 3T cannot substitute for all the information that a specialized pathologist can offer from a detailed examination of an early gestational age fetus. Nonetheless, in case the parental couple refuses the classic autopsy, taking into account the very small number of false negative results obtained, pm-MRI may be the only solution that can be offered to obtain information that may explain fetal death.

For the validation of virtual autopsy using pm-MRI at 3T in the clinical setting extensive studies are needed. The present research has the merit of analyzing the diagnostic accuracy of a clinical used scanner with a higher magnetic field for structural defects diagnosis in a little analyzed population of the fetuses resulted from termination of pregnancy. The current study also highlights new potential applications of virtual autopsy in fetuses and also brings a new perspective for postmortem imaging utility.

## Conclusions

The results of this study underline the potential diagnostic value of virtual autopsy as an alternative method for non-forensic evaluation of second trimester fetuses resulted from termination of pregnancy, with high specificity and negative predictive value. Based on our experience, virtual autopsy can be recommended as a first-line investigation for the confirmation of major structural cerebral and renal abnormalities previously detected using prenatal sonography.
